# The Real Lack in Hospital Finance

**Published:** 1920-03-13

**Authors:** 


### ENCOURAGING PROGRESS AT GLASGOW.

### The Real Lack in Hospital Finance.

The voluntary hospitals have nowhere been in greater difficulties than in Glasgow. It is, therefore, pleasant to record hopeful progress there. A very remarkable meeting of the Glasgow working men was held in February. Their representatives were keenly interested, and we have good ground for believing that their subscriptions will be doubled, perhaps more than doubled. The Western Infirmary has been to the fore in this connection, but we hope that its sister hospitals will follow suit. If they are not behind the Western in good news as we fear, we shall be glad to be corrected. Fortunately the Glasgow papers, in this, unlike many London dailies, go to the men who know what the voluntary system means for their information. We noticed in particular a well-informed article from a correspondent in the *Glasgow Herald* of February 21. After remarking that every enterprise shared the difficulties which the voluntary hospitals at present feel, the writer continued: "I believe there is ample evidence to show that voluntary means have not been exhausted." Of the numbers of people who do *not* subscribe to hospitals, the majority he declares, have never been methodically approached. Three suggestions are then made. The chief is that workmen's representatives as hospital committees should call a meeting of delegates and ask them to adopt a system of weekly contributions *on a graduated scale*, according to the wages earned. Another suggestion is that contributions from in-patients should be organised on a scale also, and the scale suggested is (for those able to give) 9s. per day for every day under treatment. Thus accident and emergency cases, who stay perhaps a few days only, would have a standard to which their generosity might conform.

Further proposals are to ask insured persons *who have no dependants* to give their insurance benefit to the hospital during the time when they are under treatment there. Insurance companies should be approached on the same lines to subscribe in proportion to the work done for their members. The establishment of wards for paying patients is also suggested.

The writer is careful to point out that the value of his suggestions does not lie in their novelty, but that it does lie in the methodical application of methods as familiar in theory as they are but lazily applied in practice. That is an excellent point. If he had only added a plea for the organisation on an equally methodical scale of an Employers' Subscription Fund, and had made an attempt to suggest how to change the casual body of annual subscribers into a self-conscious organisation with an *esprit de corps* of its own, he would have done much to complete his suggestions. But his main point should not be missed, and that is that the problem of the moment is not so much to develop or invent new sources of income as to make the most by methodical work of the old.

